# Segmentation-based cardiomegaly detection based on semi-supervised estimation of cardiothoracic ratio

**DOI:** 10.1038/s41598-024-56079-1

**Published:** 2024-03-08

**Authors:** Patrick Thiam, Christopher Kloth, Daniel Blaich, Andreas Liebold, Meinrad Beer, Hans A. Kestler

**Affiliations:** 1 Institute of Medical Systems Biology, Albert-Einstein-Allee 11, 89081 Ulm, Germany; 2 Department of Diagnostic and Interventional Radiology, Ulm University Medical Center, Albert-Einstein-Allee 23, 89081 Ulm, Germany; 3 Department of Cardiothoraxic and Vascular Surgery, Ulm University Medical Center, Albert-Einstein-Allee 23, 89081 Ulm, Germany

**Keywords:** Chest X-ray, Cardiomegaly, Deep learning, Semi-supervised learning, Semantic segmentation, Cardiology, Image processing, Machine learning, Cardiology, Image processing, Machine learning

## Abstract

The successful integration of neural networks in a clinical setting is still uncommon despite major successes achieved by artificial intelligence in other domains. This is mainly due to the black box characteristic of most optimized models and the undetermined generalization ability of the trained architectures. The current work tackles both issues in the radiology domain by focusing on developing an effective and interpretable cardiomegaly detection architecture based on segmentation models. The architecture consists of two distinct neural networks performing the segmentation of both cardiac and thoracic areas of a radiograph. The respective segmentation outputs are subsequently used to estimate the cardiothoracic ratio, and the corresponding radiograph is classified as a case of cardiomegaly based on a given threshold. Due to the scarcity of pixel-level labeled chest radiographs, both segmentation models are optimized in a semi-supervised manner. This results in a significant reduction in the costs of manual annotation. The resulting segmentation outputs significantly improve the interpretability of the architecture’s final classification results. The generalization ability of the architecture is assessed in a cross-domain setting. The assessment shows the effectiveness of the semi-supervised optimization of the segmentation models and the robustness of the ensuing classification architecture.

## Introduction

Cardiomegaly refers to an abnormally enlarged heart, that can be caused by various medical conditions such as kidney diseases, heart valve diseases, hypertension, coronary artery or pulmonary diseases, and cardiomyopathy^1^. In a clinical setting, the cardiothoracic ratio (CTR)^2^ of a posteroanterior (PA) chest radiograph constitutes a simple and useful screening technique to detect cardiomegaly. The CTR is defined as the ratio of maximum horizontal cardiac diameter by the maximum horizontal thoracic diameter, with a value higher than 0.50 usually pointing at a case of cardiomegaly (even though in some cases this specific threshold can be set to 0.55^3,4^). Expert knowledge is therefore needed for the computation and interpretation of the cardiothoracic ratio. Thus, the whole process can be time consuming and the resulting interpretation can be very subjective and significantly vary across different experts. Hence, in order to significantly improve the efficiency of the calculation process, as well as reducing the discrepancy across the interpretation of the cardiothoracic ratio, several approaches have been proposed with the main goal of performing an automatic detection of cardiomegaly based on posteroanterior chest radiographs. These approaches can be grouped in two main categories: classification-based cardiomegaly detection approaches and segmentation-based cardiomegaly detection approaches.

Classification-based cardiomegaly detection approaches rely on image-level labeled chest X-ray (CXR) images in order to generate classification models to perform the detection of cases of cardiomegaly. In most cases, transfer learning is applied in order to adapt a model pre-trained on a specific data set to the classification task at hand: Candemir et al.^5^ perform the comparison of several models that have either been fine-tuned by a limited number of CXR samples or pre-trained using a larger amount of labeled data to perform the detection of cardiomegaly instances; Zhou et al.^6^ propose an architecture consisting of three pre-trained and distinct deep neural networks: the feature representations extracted by the models are merged and subsequently used to train a neural network performing the classification task; other authors such as Bougias et al.^7^, Cardenas et al.^8^, use a specific pre-trained model to extract specific feature representations from the radiographs, which are subsequently used for the optimisation of the classification model. Even though, the results presented in these studies are very promising, most of the proposed approaches have been assessed on a single data set. A steep performance decline can be observed when such approaches are applied in a cross-domain setting^9^, thus, domain adaptation^10^ is required in order to adapt the trained models to data sets, stemming from domains other than that of the data set used to optimise the classification models. Moreover, the interpretability of the generated results requires the application of other statistical visualisation approaches such as the t-Distributed Stochastic Neighbor Embedding (t-SNE)^11^ (which interpretation can be confounding at times) or the Gradient-Weighted Class Activation Mapping (Grad-CAM)^12^.

Segmentation-based cardiomegaly detection approaches rely on pixel-level labeled chest X-ray images in order to perform the classification task. More specifically, such approaches are based on models that are optimised to perform the segmentation of both cardiac and thoracic areas. Based on the generated segmentation outputs, a CTR score is computed and the corresponding image is classified as an instance of cardiomegaly based on a specific threshold. The overall performance of such a classification architecture rests upon the accuracy of the performed cardiac and thoracic segmentation. Such an approach is adopted by Que et al.^13^, with the proposed deep neural network CardioXNet, as well as Jafar et al.^14^, with the proposed deep neural network CardioNet. Lee et al.^15^ propose and evaluate two segmentation-based approaches for the detection of cardiomegaly in CXRs: one with the segmentation models consisting of the standard U-Net architecture from Ronneberger et al.^16^ and the other consisting of segmentation models based on the XLSor model from Tang et al.^17^. Saviroonporn et al.^18^ assess four different U-Net based models for the automatic measurement of the cardiothoracic ratio. Sogancioglu et al.^19^ perform a comparison between segmentation-based and classification-based cardiomegaly detection architectures. The proposed segmentation-based approach also relies on a U-Net architecture, while the classification-based approach consists of a transfer learning method. The reported results show that the segmentation-based method significantly outperforms the classification-based method. However, in this case also, most of the approaches have been assessed based on a single data set or a combination of several data sets. The assessment of the proposed architectures in a cross-domain setting has not been performed, thus, the generalisation ability of the methods can not be determined. Moreover, most of the segmentation models are optimised in a supervised manner. Since a huge amount of annotated data is needed in order to perform the optimisation of a performant deep neural network, most of the proposed architectures rely on segmentation models that are suited for small-sized labeled data (e.g. U-Net).

In the specific case of chest radiographs, a huge amount of pixel-level annotated images is not available. Such an endeavour is time consuming and necessitates expert knowledge for the accurate pixel-level annotation. In contrast to the scarcity of huge amount of pixel-level labeled CXRs, there is a gradually increasing amount of corpora, each comprising a significantly large amount of unlabeled chest X-ray images (most of these images are labeled at the image-level but not at the pixel-level, or in other words, no manually generated segmentation masks are available). Several studies have shown that the performance of a chest X-ray segmentation model can be significantly improved by performing the optimisation of the model’s parameter in a semi-supervised setting (hence, taking advantage of a significantly larger amount of unlabeled data to improve the performance of the segmentation model), thus reducing the costs of manual annotation and generating more accurate segmentation outputs. Bortsova et al.^20^ propose a semi-supervised learning segmentation approach consisting of generating segmentation consistent output under a given set of transformations applied on both labeled and unlabeled CXRs. Wang et al.^21^ propose an improved Generative Adversarial Network (GAN) segmentation model named U-Shaped GAN for the semi-supervised segmentation of lungs from chest radiographs. The proposed model is characterised by a U-Net model in place of the usual discriminator specific to GAN models, which generates a segmentation output as well as an additional pixel-level label indicating if the generated pixel-output stems from a real or fake image. The whole architecture is trained in a semi-supervised manner by using a loss function consisting of the sum of a supervised loss computed by using the labeled data, and an unsupervised loss computed by using the unlabeled data. Brioso et al.^22^ propose a semi-supervised segmentation approach consisting of using available information regarding specific anatomical structures to guide the segmentation process when the ground truth segmentation mask for a given structure is not available. Even though the reported results in all of the previous works are very promising, the performance evaluation is performed in each study on a single data set by performing a 5-fold cross-validation evaluation. In this case also, the true generalisation ability of the proposed approaches can not determined.

Two main goals are followed in the current work: the optimisation of an effective cardiomegaly detection architecture and the traceability of the corresponding classification output (interpretable results). Therefore, the optimisation and evaluation of the segmentation-based cardiomegaly detection architecture is performed in a cross-domain evaluation setting. The goal of such a performance assessment is to evaluate the true generalisation ability of the designed classification approach, as well as its robustness in the presence of domain shift. Due to the aforementioned scarcity of a huge amount of labeled data, the optimisation of the segmentation models, upon which the whole architecture is based, is performed in a semi-supervised manner by applying a cross-consistency training approach^23^. The assessment of the performance of the designed architecture is compared to the one of an architecture consisting of models trained in a supervised manner. Moreover, the performance of the proposed architecture is also compared to that of a classification-based cardiomegaly detection architecture. Moreover, the visualisation of the segmentation output represents a feature of upmost importance, especially in a clinical setting, for the interpretability of the generated classification results.

The remainder of the work is organized as follows. In the “Materials and methods” section, a description of the data sets involved in the current study is provided, followed by a thorough description of the segmentation-based cardiomegaly detection architecture. A description of the experimental settings, as well as the performed experiments with the corresponding results is subsequently provided in the “Experimental settings and results” section. Next, a discussion of the depicted results is provided in the “Discussion” section, before the work is finally concluded in the “Conclusion” section, with a brief summary of the main findings of the study, as well as an outlook on potential future works.

## Materials and methods

In the current work, the assessment of the proposed cardiomegaly detection architecture is performed in a cross-domain setting. More specifically, the overall generalisation ability of the designed classification architecture is assessed by performing the optimisation of a model using a specific data set (referred to as training set), and subsequently evaluating the optimised model on additional data sets stemming from different domains (other than the one specific to the training set). In the specific case of cardiomegaly detection from chest X-ray images, this can be done by using a data set stemming from a specific institution to optimise the model, and performing the subsequent evaluation of the trained model on data stemming from different institutions or collected using different protocols and hardware. This form of evaluation, although particularly challenging due to the difference between the data distribution of the training set and the one of the evaluation set (also known as domain shift), constitutes a rather robust assessment of the generalization ability of the designed and optimised classification architecture. In the following, a description of the specific data sets used throughout the current work is provided, followed by a thorough description of the designed cardiomegaly detection architecture.

### Chest X-ray data sets

The *Japanese Society of Radiological Technology* (JSRT) database^24^ is a publicly available data set consisting of a total of 247 posteroanterior (PA) chest radiographs (100 with malignant pulmonary nodules, 54 with benign pulmonary nodules and 93 without a nodule) of $2048 \times 2048$ pixels resolution with a 0.175 millimeter (mm) pixel-size and a 12-bit depth, collected from 13 medical centers in Japan and 1 additional institution in the United States. Manually generated segmentation masks for the lungs, heart and clavicles, for each single image are provided by the Segmentation in Chest Radiographs (SCR) database^25^. Thus, the JSRT database is primarily used to optimise and assess multi-organs or single-organ segmentation models in a supervised learning setting^26,27^.

The publicly available *Pathology Detection in Chest Radiographs* (PadChest) data set^28^ consists of a total of 160, 868 radiographs, stemming from 67, 625 patients and recorded at the San Juan Hospital in Spain between 2009 and 2017. In contrast to the JSRT data set, no segmentation mask is available for the PadChest data set. Instead, the radiographs are annotated into a total of 170 distinct categories of radiographic findings (image-level labels), including cardiomegaly. The data set comprises chest X-ray images recorded in six different positions, including standing posteroanterior (PA) and lateral (L) views, anteroposterior (AP) supine and erect views, lordotic and oblique sternum views. Around $27\%$ of the entire data set was manually annotated by trained physicians and cases where no anomalies were found were subsequently annotated as normal. The remaining $73\%$ of the data set was automatically annotated using an attention-based recurrent neural network (trained using the set of manually annotated X-ray images). The experiments in the current work are carried out based uniquely on the manually annotated radiographs recorded in a standing posteroanterior view. Furthermore, since the current study focuses on the detection of cases of cardiomegaly, the optimisation process is performed based on image samples labeled either as cases of cardiomegaly or as normal.

The *Indiana University chest X-ray Collection* (CXR OpenI)^29^ is a publicly available data set, consisting of around 7470 manually labeled chest X-ray images, recorded in both lateral and posteroanterior views and stemming from various hospitals of the Indiana University School of Medicine. The data set is extracted from the National Library of Medicine (NLM) using the Open Access Biomedical Search Engine (OpenI)^30^. Similarly to the PadChest data set, there is no segmentation mask available for the data set. Furthermore, the data retrieved for the assessment of the proposed cardiomegaly detection architecture consist of chest X-ray images recorded in a posteroanterior position and labeled either as cases of cardiomegaly or as normal.

A custom data set (CXR Ulm) consisting of manually annotated posteroanterior radiographs stemming from a total of 131 patients (31 female and 100 male) and collected within a study at the Department of Diagnostic and Interventional Radiology of the Ulm University Medical Center in Germany, is also used for the assessment of the proposed cardiomegaly detection architecture. The data stems from a study which was (i) approved by the Ethics Commitee of the local Medical Faculty and the University Hospital (Confirmation number 115/21) and was also (ii) compliant with regards to the Health Insurance Portability and Accountability Act (HIPAA) and conducted in accordance with the Declaration of Helsinki. Additionally, informed consent was waived by the local Ethics Commitee based on the retrospective nature of study. The annotation of the data set was performed by two trained radiologists, who not only provided a label for the detected pathology but also segmentation masks for both lungs and cardiac organs. The chest X-ray images were labeled as cases of cardiomegaly based on computed cardiothoracic ratios, with a fixed threshold of 0.55.

The *National Institutes of Health Chest X-Ray Database* (CXR NIH)^31^ is a publicly available data set consisting of around 112, 120 chest X-ray images, stemming from 30, 805 patients and automatically annotated using different Natural Language Processing (NLP) techniques into either one or several categories of a total of 14 thoracic pathologies (including cardiomegaly). In cases where no pathologies were reported, the corresponding images were labeled as normal. There are also no segmentation masks available for this specific data set. Analogously to the previous data sets (PadChest, CXR OpenI, CXR Ulm), assessment experiments are performed using uniquely chest X-ray images belonging to both cardiomegaly and normal classes, and collected in a posteroanterior view. Therefore, all images belonging to the category of cardiomegaly are selected, and the same amount of images is randomly selected from the set of images labeled as normal, in order to form the assessment set specific to this data set. A summary of the data distribution specific to each of these data sets is displayed in Table 1.

**Table 1:** Data distribution.

	CXR PadChest	CXR OpenI	CXR Ulm	CXR NIH
Cardiomegaly	2140	334	79	1563
Normal	8708	1396	52	1563
Total	10, 848	1730	131	3126

Number of image samples specific to each class, for each of the data sets.

During the assessment of the proposed architecture, both JSRT and PadChest data sets are used as training sets, while the optimised architecture is subsequently evaluated on each of the remaining sets (CXR OpenI, CXR Ulm, CXR NIH). None of the samples specific to these evaluation sets are seen during the parameter optimisation of the classification architecture.

### Methodology

**Figure 1:**
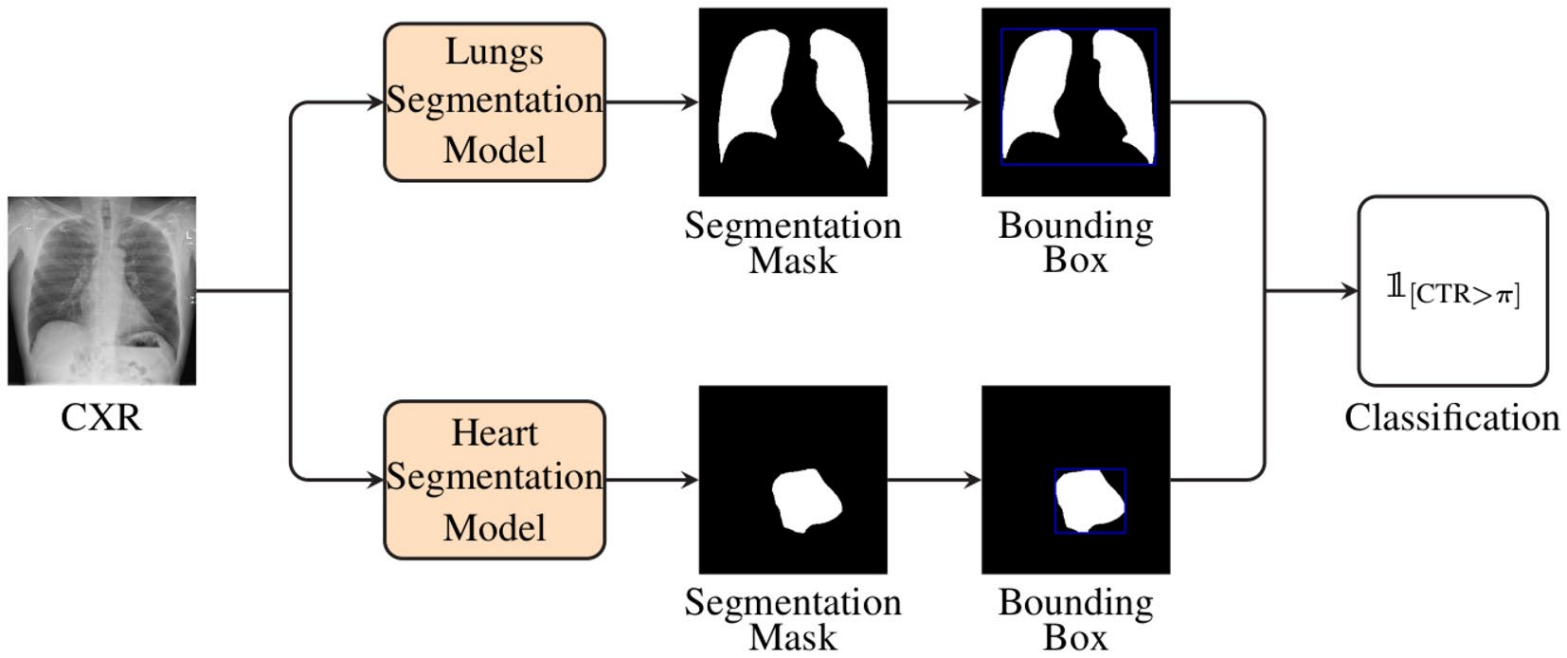
Segmentation based cardiomegaly detection architecture. Two distinct segmentation models are applied on an input image to perform the segmentation of both cardiac and lungs’ areas. Bounding boxes around the areas of interest are subsequently computed based on the resulting segmentation masks. The CTR score is calculated based on the widths of the respective cardiac and lungs’ bounding boxes. Based on a fixed threshold ($\pi$) and the computed CTR score, the input image is finally classified either as a case of cardiomegaly or as normal.

The cardiomegaly detection architecture presented in the current work consists of a segmentation-based classification approach. As depicted in Fig. 1, the architecture comprises two distinct models (which are basically two neural networks), optimised to perform the segmentation of both lungs and heart areas respectively. Given an input image, each model generates a segmentation mask of the corresponding area of interest.

Bounding boxes around the resulting areas of interest are subsequently computed, followed by the computation of the corresponding cardiothoracic ratio (CTR) based on the widths of both bounding boxes. Finally, based on a specific threshold ($\pi$), the input image is classified either as a case of cardiomegaly ($\text {CTR} > \pi$) or as normal ($\text {CTR} \le \pi$), as described in Equation 1:

1 $$\begin{aligned} \text {Output} = {\left\{ \begin{array}{ll} \text {Cardiomegaly} \, &{} \text {if} \, {\mathbf {{1}}}_{{\left[ \text {CTR} > \pi \right] }}\left( \text {CXR}\right) = 1\\ \text {Normal}  &{} \text {otherwise} \end{array}\right. } \end{aligned}$$

The overall performance, as well as the generalization ability of this specific classification architecture is inherently bound to the capacity of both models to accurately perform the segmentation of the corresponding areas of interest. In other words, the more accurate the resulting segmentation masks, the higher the classification performance of the architecture. Thus, a huge amount of annotated data is needed in order to perform some optimal optimisation of both segmentation models. In a supervised learning setting, each model is optimised based on a labeled set of images, consisting of chest X-ray images with the corresponding manually generated segmentation masks (pixel-level labels). However, manually annotated segmentation data for chest X-ray images are rather scarce (since such an annotation process is costly and time consuming), in contrast to the abundance of unlabeled chest X-ray data. Therefore, the optimisation of both segmentation models is performed in a semi-supervised learning setting, where a model is optimised based on two specific sets of data:

1. a set of labeled data ${\mathscr {X}}^{l}=\{\left( x^{l}_{1},y^{l}_{1}\right) ,\ldots ,\left( x^{l}_{n},y^{l}_{n}\right) \}$ (where $x^{l}_{i} \in \left[ 0, 255\right] ^{\text {W} \times \text {H} \times \text {C}}$ corresponds to the *i*-th chest X-ray image with a width $\text {W}$, a height $\text {H}$ and a total of $\text {C}$ channels, and the corresponding pixel-level label $y^{l}_{i} \in \left[ 0, 1\right] ^{\text {W} \times \text {H}}$, with 0 corresponding to pixels specific to the background and 1 depicting pixels of the area of interest).
2. a significantly larger set of unlabeled data ${\mathscr {X}}^{u}=\{x^{u}_{1},\ldots ,x^{u}_{m}\}$ (with $n \ll m$).

**Figure 2:**
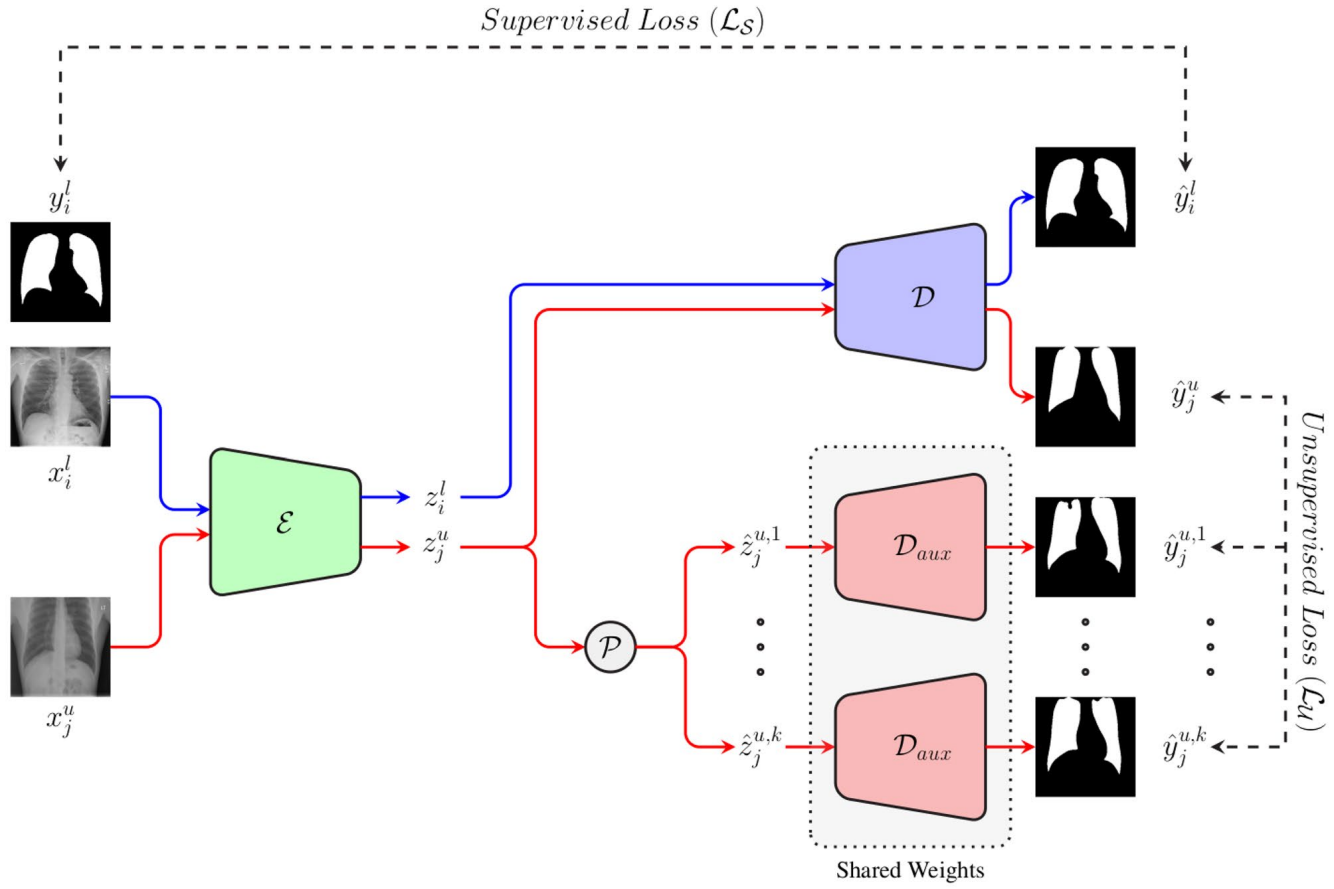
Semi-supervised segmentation approach. The architecture consists of an encoder ${\mathscr {E}}$, a main decoder ${\mathscr {D}}$ and an auxiliary decoder ${\mathscr {D}}_{aux}$. During each iteration, labeled samples ($x_{i}^{l}$) and unlabeled samples ($x_{j}^{u}$) are fed into the shared encoder. The resulting representations ($z_{i}^{l}$ and $z_{j}^{u}$) are subsequently fed into the main decoder, which generates the segmentation masks for both labeled and unlabeled images (${\hat{y}}_{i}^{l}$ and ${\hat{y}}_{j}^{u}$). Concurrently, a set of *k* distinctive perturbations (${\mathscr {P}}$) are applied to the latent representations specific to the unlabeled samples ($z_{j}^{u}$), and the resulting representations ($\{{\hat{z}}_{j}^{u,d}\}_{1 \le d \le k}$) are fed into the auxiliary decoder. The resulting set of auxiliary segmentation masks ($\{{\hat{y}}_{j}^{u,d}\}_{1 \le d \le k}$) are used in combination with the corresponding output of the main decoder (${\hat{y}}_{j}^{u}$) to compute an unsupervised loss (${\mathscr {L}}_{{\mathscr {U}}}$), while the supervised loss (${\mathscr {L}}_{{\mathscr {S}}}$) is calculated based on the labeled samples’ output of the main decoder (${\hat{y}}_{i}^{l}$) and the corresponding labels ($y_{i}^{l}$).

Inspired by the work presented by Ouali et al.^23^, cross-consistency training is applied in order to perform the optimisation of a models’ parameters. An overview of the architecture and training procedure is depicted in Fig. 2. The architecture consists of a total of three neural networks: a shared encoder ${\mathscr {E}}$, a main decoder ${\mathscr {D}}$ and an auxiliary decoder ${\mathscr {D}}_{aux}$. Images stemming from both labeled and unlabeled sets are simultaneously fed into the shared encoder ${\mathscr {E}}$. The generated latent representations are subsequently fed into the two remaining neural networks. The representations specific to both labeled and unlabeled images are fed into the main decoder ${\mathscr {D}}$.

Concurrently, a set of *k* stochastic perturbations are applied on each of the representations stemming from the set of unlabeled images, and the resulting altered representations are fed into the auxiliary decoder ${\mathscr {D}}_{aux}$. Based on the output of the main decoder and the provided labels, the parameters of the main decoder are optimised using a supervised loss function ${\mathscr {L}}_{{\mathscr {S}}}$. Meanwhile, the parameters specific to the auxiliary decoder are optimised based on an unsupervised loss function ${\mathscr {L}}_{\mathscr {U}}$, computed based on its output and those from the main decoder stemming uniquely from the unlabeled samples. This is done in order to enforce a certain level of consistency between the output of the main decoder ${\mathscr {D}}$ and the auxiliary decoder ${\mathscr {D}}_{aux}$. The parameters of the shared encoder are optimised based on a weighted sum of both loss functions (${\mathscr {L}}$), as follows:

2 $$\begin{aligned} {\mathscr {L}} = {\mathscr {L}}_{{\mathscr {S}}} + \omega _{{\mathscr {U}}}{\mathscr {L}}_{{\mathscr {U}}} \end{aligned}$$

where, $\omega _{{\mathscr {U}}}$ is a weighting function specific to the unsupervised loss. By enforcing the segmentation consistency between the output of the main decoder and the one of the auxiliary decoder regarding unlabeled chest X-ray images, the representations generated by the shared encoder are further enhanced by taking advantage of additional information stemming from unlabeled samples. Following the optimisation of the models, both the shared encoder and the main decoder are used to perform the segmentation of unseen samples during inference.

In the current work, the supervised loss consists of the combination of a pixel-level classification loss and a segmentation loss as depicted in Eq. (3):

3 $$\begin{aligned} {\mathscr {L}}_{\mathscr {S}} = \frac{1}{\textrm{bs}^{l}}\sum _{i = 1}^{\textrm{bs}^{l}}\left( \mathbf {{H}}(y^{l}_{i},{\hat{y}}^{l}_{i}) + {\textrm{dice}}(y^{l}_{i},{\hat{y}}^{l}_{i})\right) \end{aligned}$$

where $\textrm{bs}^{l}$ depicts the batch size for the set of labeled samples, $\mathbf {{H}}$ represents the Binary Cross-Entropy loss (BCE) and $\textrm{dice}$ represents the Dice loss. Meanwhile, the unsupervised loss consists of the Mean Squared Error (MSE) between the output of the main decoder and those of the auxiliary decoder specific to the unlabeled set of images, as depicted in Eq. (4):

4 $$\begin{aligned} {\mathscr {L}}_{{\mathscr {U}}}= & {} \frac{1}{k \times {\textrm{bs}}^{u}}\sum _{j = 1}^{\textrm{bs}^{u}}\sum _{p = 1}^{k}{\textrm{d}}({\hat{y}}^{u}_{j}, {\hat{y}}^{u, p}_{j}) \end{aligned}$$

5 $$\begin{aligned} {\textrm{d}}({\hat{y}}^{u}_{j},{\hat{y}}^{u,p}_{j})= & {} \sum ^{\text {W}}_{w = 1}\sum ^{\text {H}}_{h = 1} \left( {\hat{y}}^{u}_{j}\left( w, h\right) - {\hat{y}}^{u,p}_{j}\left( w, h\right) \right) ^{2} \end{aligned}$$

where $\textrm{bs}^{u}$ depicts the batch size for the set of unlabeled samples and ${\textrm{d}}({\hat{y}}^{u}_{j},{\hat{y}}^{u,p}_{j})$ (see Eq. 5) represents the pixel-level squared error between both outputs ${\hat{y}}^{u}_{j}$ (from the main decoder ${\mathscr {D}}$) and ${\hat{y}}^{u,p}_{j}$ (from the auxiliary decoder ${\mathscr {D}}_{aux}$). The weighting function $\omega _{{\mathscr {U}}}$ specific to the unsupervised loss corresponds to a Gaussian ramp-up function (see Eq. 6) as proposed by Laine and Aila^32^:

6 $$\begin{aligned} \omega _{{\mathscr {U}}}\left( t\right) = {\left\{ \begin{array}{ll} \exp {\left( -5\left( 1 - \frac{t}{L}\right) ^2\right) } &{} \text {if} \, t < L \\ 1 &{} \text {otherwise} \end{array}\right. } \end{aligned}$$

where *t* depicts the current optimisation epoch and *L* depicts the ramp-up length. The computed unsupervised loss weight slowly ramps up from 0 to 1 during the optimisation process, therefore reducing the impact of noisy segmentation outputs of the main decoder ${\mathscr {D}}$ during the early phase of the optimisation process. The perturbations applied to the latent representations specific to the unlabeled images consist of feature based perturbations and random perturbations (as proposed by Ouali et al.^23^). These perturbations have not just proven to be effective in such areas as semi-supervised semantic segmentation^23,33,34^ or object localisation^35^, but are also very simple to implement.

Feature based perturbations consist of injecting random noise into the latent representation stemming from the shared encoder ${\mathscr {E}}$. Two specific feature based perturbations are applied to the latent representation in the current work:

- F-Noise: $\forall j, \, z^{u,1}_{j} = z^{u}_{j} + \left( z^{u}_{j} \odot \textrm{N}\right)$ with $\textrm{N} \sim {\mathscr {U}}\left( -0.3, 0.3\right)$ being a uniformly sampled random tensor of the same shape as $z^{u}_{j}$.
- F-Drop: $\forall j, \, z^{u,2}_{j} = z^{u}_{j} \odot \mathrm {M_{drop}}$, where $\mathrm {M_{drop}}$ represents a binary tensor of the same shape as $z^{u}_{j}$ obtained based on an uniformly sampled threshold $\gamma \sim {\mathscr {U}}\left( 0.6, 0.9\right)$ and the normalized channel-wise averaged tensor of $z^{u}_{j}$, denoted $\tilde{z}^{u}_{j}$ as follows: $\mathrm {M_{drop}} = \mathbf {{1}}_{\tilde{z}^{u}_{j} > \gamma }$.

Random perturbations consist of randomly dropping some of the activations of the latent representation. In the current work, Dropout is applied to the latent representation with an uniformly sampled dropout rate $\text {r} \sim {\mathscr {U}}\left( 0.1, 0.7\right)$.

## Experimental settings and results

In the following section, a thorough description of the performed experiments is provided. First, the experimental settings are described, followed by a description and discussion of each performed experiment with its corresponding results.

### Experimental settings

Before being fed into the designed architecture, chest X-ray images are pre-processed in order to significantly reduce the amount of noise within the images and homogenize the structure of the input data across different domains. In the current work, each image is first resized to the shape $299 \times 299 \times 3$ and subsequently converted into a single-channel gray-scale image. Subsequently, Contrast Limited Adaptive Histogram Equalization (CLAHE)^36^ is applied in order to enhance the contrast of the resulting gray-scale image. Next, a three-channel image is generated by replicating the single-channel contrast enhanced image three times. And finally, the pixel values of the resulting image are normalized within the range $\left[ 0, 1\right]$, by dividing each pixel value in each of the three channels by the maximum pixel value of 255. Moreover, since the labeled set of data consists of a rather limited amount of chest X-ray images (The JSRT database consists of a total of 247 chest X-ray images with the corresponding pixel-level labels), data augmentation is performed (uniquely on the set of labeled images) by applying a set of geometrical transformations consisting of random horizontal and vertical flipping, random image rotation in a range of $\left[ 0^{\circ }, 10^{\circ }\right]$, and a $10\%$ image zoom-in. The transformations are applied on both chest X-ray images and the corresponding pixel-level labels in order to generate an increased amount of consistent labeled data.

Each segmentation model consists of an Encoder–Decoder network which takes as input a chest X-ray image and as label the corresponding pixel-level annotated image. In a semi-supervised learning setting, both decoders (${\mathscr {D}}$ and ${\mathscr {D}}_{aux}$) have an identical architecture. While the parameters of the main decoder ${\mathscr {D}}$ are optimised by using the supervised loss, the parameters of the auxiliary decoder ${\mathscr {D}}_{aux}$ are optimised by using the unsupervised loss. The parameters of the encoder ${\mathscr {E}}$ are optimised by a weighted sum of both supervised and unsupervised losses. A main component of the designed neural networks consists of a *convolutional block*, which comprises a 2-dimensional convolutional layer, followed by a Batch Normalization layer as a regularization approach and subsequently a Rectified Linear Unit (ReLU) activation function. The ensuing feature map is subsequently fed into an attention layer, consisting of the Convolutional Block Attention Module (CBAM)^37^. The designed architectures of the encoder (${\mathscr {E}}$), and both decoders (${\mathscr {D}}$, ${\mathscr {D}}_{aux}$) are depicted in Table 2.

**Table 2:** Neural networks’ architectures.

Encoder ($\varvec{{\mathscr {E}}}$)				
Layer	No. filters	Kernel size	Strides	Padding
$3 \times \text {Convolutional block}$	16	$(3 \times 3)$	$(1 \times 1)$	$\textrm{same}$
$\text {Max pooling}$	−	$(2 \times 2)$	$(2 \times 2)$	−
$3 \times \text {Convolutional block}$	32	$(3 \times 3)$	$(1 \times 1)$	$\textrm{same}$
$\text {Max Pooling}$	−	$(2 \times 2)$	$(2 \times 2)$	−
$3 \times \text {Convolutional block}$	64	$(3 \times 3)$	$(1 \times 1)$	$\textrm{same}$
$\text {Max Pooling}$	−	$(2 \times 2)$	$(2 \times 2)$	−
$3 \times \text {Convolutional block}$	128	$(3 \times 3)$	$(1 \times 1)$	$\textrm{same}$
$\text {Max Pooling}$	−	$(2 \times 2)$	$(2 \times 2)$	−
$3 \times \text {Convolutional block}$	256	$(3 \times 3)$	$(1 \times 1)$	$\textrm{same}$
$\text {Max pooling}$	−	$(2 \times 2)$	$(2 \times 2)$	−
Decoder ($\varvec{{\mathscr {D}}}$, $\varvec{{\mathscr {D}}}_{aux}$)				
Layer	No. filters	Kernel size	Strides	Padding
$3 \times \text {Convolutional block}$	256	$(3 \times 3)$	$(1 \times 1)$	$\textrm{same}$
$\text {Conv2DTranspose}$	128	$(3 \times 3)$	$(2 \times 2)$	$\textrm{same}$
$$\text {Batch normalization}$$				
$$\text {Activation: ReLU}$$				
$3 \times \text {Convolutional block}$	128	$(3 \times 3)$	$(1 \times 1)$	$\textrm{same}$
$\text {Conv2DTranspose}$	64	$(3 \times 3)$	$(2 \times 2)$	$\textrm{valid}$
$$\text {Batch normalization}$$				
$$\text {Activation: ReLU}$$				
$3 \times \text {Convolutional block}$	64	$(3 \times 3)$	$(1 \times 1)$	$\textrm{same}$
$\text {Conv2DTranspose}$	32	$(3 \times 3)$	$(2 \times 2)$	$\textrm{same}$
$$\text {Batch normalization}$$				
$$\text {Activation: ReLU}$$				
$3 \times \text {Convolutional block}$	32	$(3 \times 3)$	$(1 \times 1)$	$\textrm{same}$
$\text {Conv2DTranspose}$	16	$(3 \times 3)$	$(2 \times 2)$	$\textrm{valid}$
$$\text {Batch normalization}$$				
$$\text {Activation: ReLU}$$				
$3 \times \text {Convolutional block}$	16	$(3 \times 3)$	$(1 \times 1)$	$\textrm{same}$
$\text {Conv2DTranspose}$	1	$(3 \times 3)$	$(2 \times 2)$	$\textrm{valid}$
$$\text {Batch normalization}$$				
$$\text {Activation: ReLU}$$				
$\text {Conv2D}$	1	$(3 \times 3)$	$(1 \times 1)$	$\textrm{same}$
$$\text {Batch normalization}$$				
$$\text {Activation: sigmoid}$$				

During the optimisation phase in a semi-supervised setting, a specific batch size ($\textrm{bs}$) is set, such that $\textrm{bs} = \textrm{bs}^{l} + \textrm{bs}^{u}$: $\textrm{bs}^{l}$ represents the batch size specific to the set of labeled samples and $\textrm{bs}^{u}$ corresponds to the batch size specific to the set of unlabeled samples. Given $\textrm{bs}$, $\textrm{bs}^{l}$ and $\textrm{bs}^{u}$ are computed as depicted in Eq. (7) (where *n* is the number of labeled samples in the training set and *m* is the number of unlabeled samples in the training set) and Eq. (8). Due to memory constraints, $\textrm{bs}$ is set to 16 in the current work.

7 $$\begin{aligned} \delta= & {} \frac{\left( n + m\right) }{\textrm{bs}} \end{aligned}$$

8 $$\begin{aligned} \textrm{bs}^{l}= & {} \left\lfloor \frac{n}{\delta } \right\rfloor \, \text {and} \, \textrm{bs}^{u} = \lceil \frac{m}{\delta } \rceil \end{aligned}$$

Moreover, the optimisation is performed with a fixed learning rate set empirically to $10^{-3}$ for a total of 100 epoches. The ramp-up length $\textrm{L}$ (see Eq. (6)) is set to 50. The optimiser used throughout the current work consists of the Adaptive Moment Estimation optimisation algorithm (Adam)^38^. During the optimisation phase, $20\%$ of the set of labeled samples are used as validation set and the remaining $80\%$ is used as training set. Concerning the set of unlabeled samples, $10\%$ is used as validation set and the remaining $90\%$ as training set. The Jaccard index (see Eq. (9)) is used as segmentation performance evaluation metric:

9 $$\begin{aligned} J\left( y, {\hat{y}}\right) = \frac{|y \cap {\hat{y}}|}{|y \cup {\hat{y}}|} \end{aligned}$$

where *y* represents a pixel-level annotated image (ground truth) and ${\hat{y}}$ the output of the decoder (prediction). Following the optimisation of both segmentation models (one for the heart and the other for both lungs), the performance of the cardiomegaly detection architecture (see Fig. 1) is assessed based on the following performance assessment metrics:

- $$\text {Sensitivity}\,\left( \text {Sens}\right) $$:   $$\frac{\text {TP}}{\text {TP}+\text {FN}}$$
- $$\text {Specificity}\,\left( \text {Spec}\right) $$:   $$\frac{\text {TN}}{\text {TN}+\text {FP}}$$
- $$\text {Geometric Mean}\,\left( \text {G-Mean}\right) $$: $$\sqrt{\text {Sens}\times \text {Spec}}$$
- $$\text {Accuracy}\,\left( \text {Acc}\right) $$:   $$\frac{\text {TP} + \text {TN}}{\text {TN} + \text {FP} + \text {TP} + \text {FN}}$$

with $\text {TP}$: True Positives; $\text {FN}$: False Negatives; $\text {TN}$: True Negatives; $\text {FP}$: False Positives.

The Area Under the Receiver Operating Characteristic (ROC) Curve (AUC) is also used as an additional performance evaluation metric. Thereby, cases of cardiomegaly are set as belonging to the positive class (positive instances), while normal samples are set as belonging to the negative class (negative instances) throughout the entirety of the performed experiments. All implementations and evaluations performed in the current work were done with the libraries Tensorflow^39^, Keras^40^, and Scikit-learn^41^. The optimisation process of each segmentation model was performed on a single Tesla V100 SXM2 Graphics Processing Unit (GPU) with 32 gigabytes of memory, with the Compute Unified Device Architecture (CUDA) version 11.4. The inference was subsequently performed on a M1 Macbook Pro with 16 gigabytes of memory.

### Segmentation-based cardiomegaly detection

**Figure 3:**
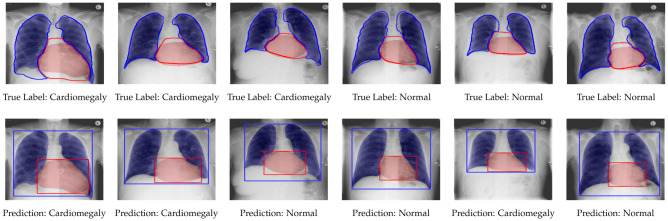
Semi-supervised segmentation results (CXR Ulm). The top row consists of the segmentation models’ output (filled areas) and the ground truth (contours). At the bottom, the exact same set of images is displayed as above, this time however uniquely with the segmentation model’s output and the computed bounding boxes around the areas of interest.

The first experiment consists of assessing the performance of the proposed segmentation-based cardiomegaly detection architecture in both supervised and semi-supervised settings, and comparing the performance of each optimisation approach based on the data sets CXR Ulm, CXR OpenI, and CXR NIH. In order to perform the classification task, a threshold of $\pi = 0.55$ is used for the CXR Ulm data set, which is the same threshold used by the physicians who performed the annotation of the data set. For both CXR OpenI and CXR NIH data sets, the threshold is set as follows: $\pi = 0.50$. None of the images specific to these data sets have been seen during the optimisation process of the segmentation models. Even though such an assessment is rather challenging, it usually depicts the true generalisation ability of the proposed classification approach. In a supervised learning setting, a segmentation model consists uniquely of the encoder ${\mathscr {E}}$ and the main decoder ${\mathscr {D}}$. Its optimisation is performed based uniquely on the set of labeled samples (the JSRT data set) and also by using uniquely the supervised loss (${\mathscr {L}}_{{\mathscr {S}}}$). The remaining optimisation parameters are the same as in the case of the semi-supervised model optimisation. In a semi-supervised learning setting, the architecture is as described in Fig. 3 and the set of labeled instances consists of the JSRT data set, while the set of unlabeled instances consists of the PadChest data set. The results of the cardiomegaly detection task, based on segmentation models trained in both supervised and semi-supervised settings are depicted in Table 3.

**Table 3:** Cardiomegaly detection performance.

CXR Ulm					
Segmentation approach	Sensitivity (%)	Specificity (%)	G-Mean (%)	Accuracy (%)	AUC (%)
Supervised segmentation	56.96	86.54	70.21	68.70	71.75
Semi-supervised segmentation	$\mathbf {93.67}$	$\mathbf {90.38}$	$\mathbf {92.01}$	$\mathbf {92.37}$	$\mathbf {92.03}$
CXR OpenI					
Segmentation approach	Sensitivity	Specificity	G-Mean	Accuracy	AUC
Supervised segmentation	81.74	66.40	73.67	69.36	74.07
Semi-supervised segmentation	$\mathbf {90.12}$	$\mathbf {80.80}$	$\mathbf {85.33}$	$\mathbf {82.60}$	$\mathbf {85.46}$
CXR NIH					
Segmentation approach	Sensitivity	Specificity	G-Mean	Accuracy	AUC
Supervised segmentation	79.40	66.79	72.82	73.10	73.10
Semi-supervised segmentation	$\mathbf {88.87}$	$\mathbf {75.75}$	$\mathbf {82.05}$	$\mathbf {82.31}$	$\mathbf {82.31}$

The numbers in bold depict the best overall performance across all evaluated approaches.

At a glance, the architecture consisting of segmentation models optimised in a semi-supervised learning setting systematically outperforms the one based on models optimised in a supervised learning setting for all data sets. Thus, the output of a model trained in a semi-supervised manner is more accurate than the one of a model trained in a supervised manner. This is also confirmed by the segmentation performance on both lungs and heart areas for the CXR Ulm data set: in a supervised learning setting the lungs’ segmentation model achieves an averaged Jaccard score of $86.07\%$, while the heart’s segmentation model achieves an averaged Jaccard score of $65.38\%$; in a semi-supervised learning setting however, the lungs’ segmentation model achieves an averaged Jaccard score of $89.88\%$, while the heart’s segmentation model achieves an averaged Jaccard score of $76.87\%$. The cross-consistency training approach successfully extracts meaningful information from a set of unlabeled samples in order to enhance the latent representation stemming from the decoder and therefore significantly improves the resulting segmentation output. Learning uniquely from a significantly smaller set of labeled data leads to sub-optimal segmentation results, even after the application of data augmentation. Additionally, while considering the depicted classification results, one can see that the designed cardiomegaly detection architecture performs rather well in a cross-domain setting, since good classification performances can be observed across all the data sets. Thus, the designed architecture based on segmentation models trained in a semi-supervised manner exhibits a good generalisation ability. Since the labels specific to the CXR Ulm data set are available, a visualization of some of the segmentation outputs with models trained in a semi-supervised manner is depicted in Fig. 3.

The color blue is specific to the lungs while the color red refers to the heart. The displayed contours constitute the ground truth (or manually generated segmentation results), while the filled areas constitute the segmentation models’ output (depicted in the top row of Fig. 3). Furthermore, the resulting bounding boxes, based on the segmentation models’ output are also displayed (in the bottom row of Fig. 3). While the lungs’ can be relatively well segmented, the heart area revealed to be rather challenging. The segmentation model specific to the heart had more difficulties in performing an accurate segmentation in several cases. It was also observed that most of the occurred miss-classifications were due to an inaccurate segmentation of the heart. Therefore, it is believed that an improvement of the heart segmentation model should also result in an improvement of the overall performance of the cardiomegaly detection architecture.

### Classification- vs. segmentation-based cardiomegaly detection

The next experiment consists of comparing the performance of the segmentation-based cardiomegaly detection approach, to the one of a classification-based cardiomegaly detection approach. Based on the work presented by Thiam et al.^9^, a model is trained using uniquely the PadChest data set with the corresponding image-level labels (one-hot encoding consisting of $\left( 1,0\right)$ for normal and $\left( 0, 1\right)$ for cardiomegaly) in order to generate a classification-based cardiomegaly detection model using a transfer learning approach. The architecture of the model comprises a backbone consisting of pre-trained convolutional layers, followed by an additional and single trainable convolutional layer and a subsequent global average pooling (GAP) layer. The backbone is generated by removing the top fully connected (FC) layers of a pre-trained deep neural network and freezing the remaining convolutional layers.

**Table 4:** Classification approach vs. segmentation approach.

Detection approach	CXR Ulm (%)	CXR OpenI (%)	CXR NIH (%)
Classification (transfer learning)	53.37	82.78	59.18
Supervised segmentation	70.21	73.67	72.82
Semi-supervised segmentation	$\mathbf {92.01}$	$\mathbf {85.33}$	$\mathbf {82.05}$

The performances are depicted in terms of geometric mean (G-Mean). The numbers in bold depict the best overall G-Mean performance across all evaluated approaches.

Significant values are in bold.

**Figure 4:**
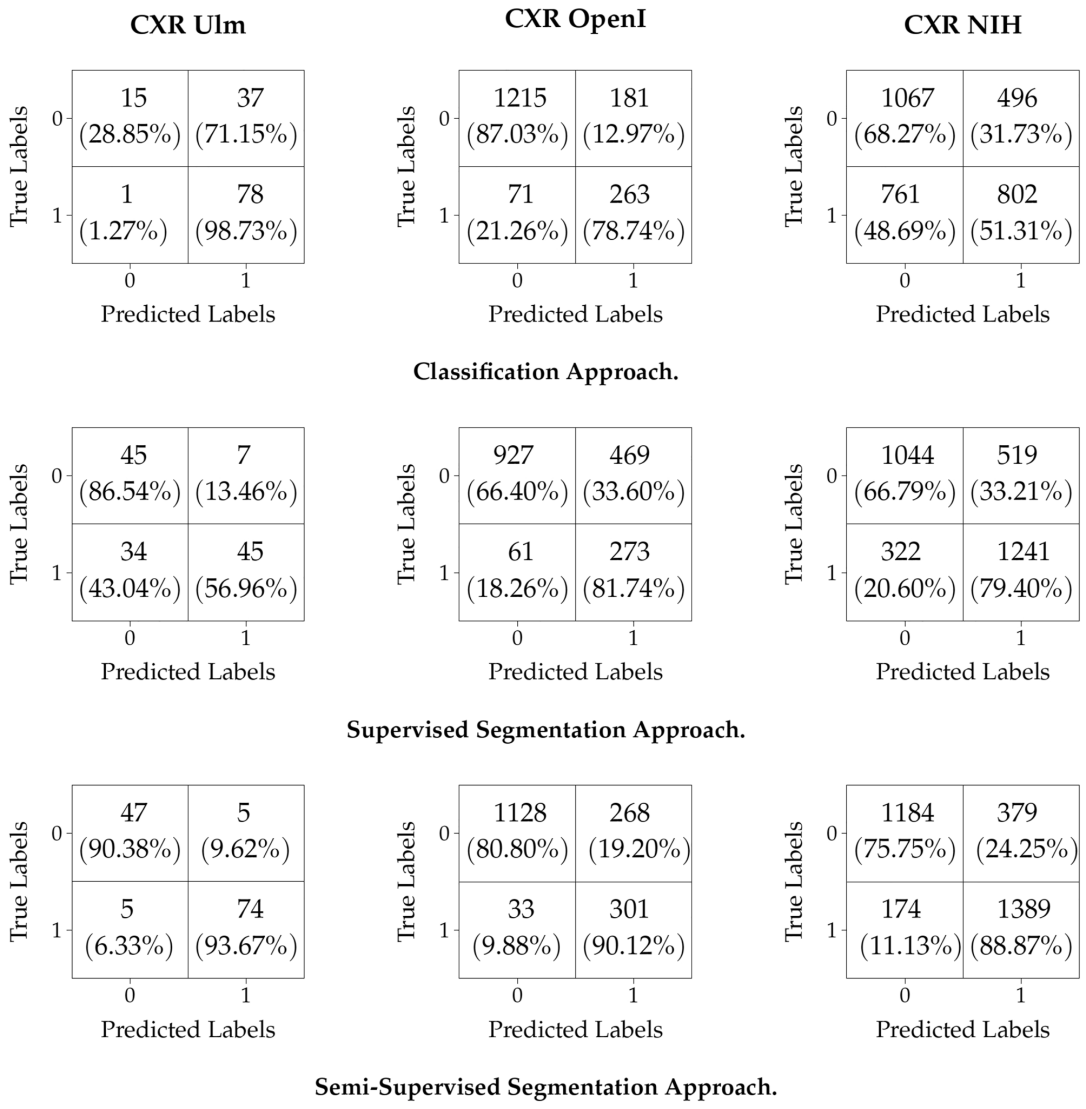
Confusion matrices. The label 0 corresponds to normal CXR images, while the label 1 corresponds to cases of cardiomegaly.

For the current experiments, the backbone consists of the InceptionV3 model^42^ trained on the ImageNet database^43^. Subsequent layers are added on top of the backbone: first a trainable convolution layer consisting of 1024 filters ($3 \times 3$ kernels and $1 \times 1$ strides), followed by a Batch Normalization layer and a subsequent Rectified Linear Unit (ReLU) activation; the output is subsequently fed into a global average pooling (GAP) layer. The resulting feature representation is subsequently fed into a classifier consisting of two subsequent fully connected layers. The first layer uses a ReLU activation function with a total of 512 units and the second layer uses a Softmax activation function with a total of 2 units to generate the final output of the classification model. Regularization is performed in this case by placing Dropout layers with a fixed dropout rate of 0.25 between both fully connected layers, as well as between the GAP layer and the first fully connected layer. During the optimisation process, a fixed learning rate of $10^{-6}$ is used and the batch size is set to 16. The optimisation process goes on for a total of 200 iterations, using the Adam optimiser. In order to account for the imbalanced data distribution within the PadChest data set, samples are weighted as follows:

10 $$\begin{aligned} \forall x^{u}_{j} \in {\mathscr {X}}^{u},\, w_{j} = {\left\{ \begin{array}{ll} \frac{m^{-}}{m} &{} \text {if} \, y_{j} = \left( 0, 1\right) \\ \frac{m^{+}}{m} &{} \text {if} \, y_{j} = \left( 1, 0\right) \end{array}\right. } \end{aligned}$$

where $m = m^{-} + m^{+}$, with $m^{-} = \Vert \{x^{u}_{j} \in {\mathscr {X}}^{u}\,|\,y_{j} = \left( 1, 0\right) \}\Vert$ and $m^{+} = \Vert \{x^{u}_{j} \in {\mathscr {X}}^{u}\,|\,y_{j} = \left( 0, 1\right) \}\Vert$. Following its optimisation, the classification model is subsequently applied on the testing sets CXR Ulm, CXR OpenI, and CXR NIH. The yielded results are summarised and depicted in Table 4, while the corresponding confusion matrices are depicted in Fig. 4.

It can be clearly seen that the segmentation-based cardiomegaly detection approach, based on segmentation models trained in a semi-supervised manner outperforms the classification-based cardiomegaly detection approach for each of the testing sets, thus further pointing to the effectiveness of the proposed detection architecture. Similar results have been presented by Sogancioglu et al.^19^, where the authors performed a comparison of both segmentation-based and classification-based cardiomegaly detection on a single data set. The results reported in that study also show that segmention-based detection approaches outperform classification-based detection approaches (even though the experiments were conducted on a single data set). Furthermore, while the impact of the domain shift can be seen from the results specific to the classification-based detection approach, both segmentation-based detection approaches (in both supervised and semi-supervised settings) prove to be more robust in this regard by yielding better classification performances for almost all of the testing sets.

### U-Net vs. semi-supervised segmentation based cardiomegaly detection

Based on the fact that most of the previous works rely on a U-Net model to perform the segmentation of specific areas of interests in a chest X-ray image, the last experiment consists of comparing the performance of the described segmentation-based cardiomegaly detection approach with segmentation models trained in a semi-supervised manner, to the one of a cardiomegaly detection approach based on U-Net models. The encoder of the U-Net models consists of the frozen pre-trained convolutional layers of an InceptionV3 model (optimised on the ImageNet data set^44^). The decoder consists of the mirrored layers of the encoder (with additional up-sampling layers), with skip connections between the corresponding layers. Each skip connection is followed by a Dropout layer with a rate fixed empirically to 0.3. The whole architecture is trained using uniquely the JSRT data set (with the corresponding segmentation masks) for a total of 100 epochs, with a fixed learning rate of $10^{-4}$. The Focal loss^45^ is used in this case to optimise the whole architecture with an Adam optimiser. The classification results depicted in Table 5 clearly show that the overall performance of the semi-supervised cardiomegaly detection architecture is substantially better than the one of the detection approach based on U-Net models. These results reinforce the previously stated assumption that the diversity of the data set used to optimise a classification model plays a crucial role in its generalisation ability, since the models trained in a semi-supervised manner are able to improve the feature representations generated by the encoder by using additional information stemming from a diverse set of unlabeled CXR images, resulting in improved segmentation outputs (and thus improved cardiomegaly detection results).

**Table 5:** U-Net approach vs. semi-supervised segmentation approach.

Detection approach	CXR Ulm (%)	CXR OpenI (%)	CXR NIH (%)
U-Net segmentation	82.46	79.44	75.00
Semi-supervised segmentation	$\mathbf {92.01}$	$\mathbf {85.33}$	$\mathbf {82.05}$

The performances are depicted in terms of geometric mean (G-Mean). The numbers in bold depict the best overall G-Mean performance across all evaluated approaches.

The best overall results are in bold.

## Discussion

The presented results clearly show that the performance of a segmentation model can be substantially improved by using information stemming from unlabeled samples. In the current work, cross-consistency training has proven to be a simple and effective semi-supervised training approach. Some significant performance improvement of the cardiomegaly detection architecture could be achieved by using models trained in a semi-supervised manner, in comparison to using models trained in a supervised manner. Thus, an effective integration of information stemming from unlabeled samples can significantly reduce the amount of labeled samples required in order to achieve high classification performances, therefore significatly reducing the costs of manual annotation. Additionally, the results of the subsequent experiments show that the proposed segmentation-based cardiomegaly detection approach outperforms the classification-based approach (based on image-level labels) in a cross-domain setting. Moreover, segmentation-based cardiomegaly detection approaches proved to be more robust than classification-based cardiomegaly detection approaches, regarding the domain shift observed while performing the detection in a cross-domain setting. Furthermore, since the output of the segmentation models can be easily plotted and visualized, the resulting classification results can be easily interpreted, therefore bringing more clarity to the generated predictions and allowing the identification of the detection architecture’s flaws and limitations. This is particularly relevant in a clinical setting, where a visualisation of the automatically generated segmentation provides more insights than the results of the classification or detection task alone. Finally, even though the experiments were performed in a challenging cross-domain setting, the yielded results point at a good generalisation ability of the proposed architecture. Previous works generally focus on single data sets and report similar results^15,19^. However, such approaches suffer from the domain shift when applied on data sets stemming from other centers, resulting in sub-optimal performances. Thus, the diversity of the data sets used to perform the optimisation of the segmentation models plays a significant role in the resulting generalisation ability of the optimised models.

## Conclusion

As a summary, cross-consistency training has proven to be very effective since the segmentation models trained in a semi-supervised setting were able to significantly improve the performance of the cardiomegaly detection architecture, in comparison to the models trained in a supervised manner. The diversity of the data sets used for the optimisation of the segmentation models positively impacted the generalisation ability of the detection architecture in a cross-domain setting. The interpretability of the generated results is further improved by the segmentation-based approaches, which is of upmost importance in a clinical setting. However, it is believed that the performance of the proposed architecture can be further improved, by enhancing the performance of the model specific to the heart area, since the observed miss-classifications were mostly due to an inaccurate segmentation output of this specific area of interest. Future directions of the current work could consist in assessing other forms of perturbations to be applied at different levels of granularity within the segmentation models, as well as an assessment of other semi-supervised learning approaches for the optimisation of the segmentation models^46^.

## Data Availability

Publicly available datasets were analyzed in this study. The *Japanese Society of Radiological Technology (JSRT) database* can be found at http://db.jsrt.or.jp/eng.php; The *Pathology Detection in Chest Radiographs (PadChest) data set* can be found at: https://bimcv.cipf.es/bimcv-projects/padchest/; The *Indiana University chest X-ray Collection (CXR OpenI)* can be found at: https://openi.nlm.nih.gov/gridquery?it=xg &coll=cxr &m=1 &n=100; The *National Institutes of Health Chest X-Ray Database (CXR NIH)* can be found at: https://nihcc.app.box.com/v/ChestXray-NIHCC. The custom *CXR Ulm* data set analysed during the current study is available from the corresponding author (MB) on reasonable request.

## References

[1] Felker GM (2000). Underlying causes and long-term survival in patients with initially unexplained cardiomyopathy. New Engl. J. Med..

[2] Danzer CS (1919). The cardiothoracic ratio: An index of cardiac enlargement. Am. J. Med. Sci..

[3] Pouraliakbar, H. Chapter 6—Chest radiography in cardiovascular disease. In *Practical Cardiology* (Maleki, M., Alizadehasl, A. & Haghjoo, M. eds.) . 113–130. 10.1016/B978-0-323-51149-0.00006-7 (Elsevier, 2018).

[4] Simkus P (2021). Limitations of cardiothoracic ratio derived from chest radiographs to predict real heart size: Comparison with magnetic resonance imaging. Insights Imaging.

[5] Candemir, S., Rajaraman, S., Thoma, G. & Antani, S. Deep learning for grading cardiomegaly severity in chest X-rays: An investigation. In *2018 IEEE Life Sciences Conference (LSC)*. 109–113. 10.1109/LSC.2018.8572113 (2018).

[6] Zhou S, Zhang X, Zhang R (2019). Identifying cardiomegaly in Chestx-ray8 using transfer learning. Stud. Health Technol. Inform..

[7] Bougias H, Georgiadou E, Malamateniou C, Stogiannos N (2020). Identifying cardiomegaly in chest X-rays: A cross-sectional study of evaluation and comparison between different transfer learning methods. Acta Radiol..

[8] Cardenas, D. *et al.* Multicenter validation of convolutional neural networks for automated detection of cardiomegaly on chest radiographs. In *Anais Principais do Simpósio Brasileiro de Computação Aplicada à Saúde (SBCAS 2020)*. 179–190. 10.5753/sbcas.2020.11512 (Sociedade Brasileira de Computação—SBC, 2020).

[9] Thiam P (2023). Unsupervised domain adaptation for the detection of cardiomegaly in cross-domain chest X-ray images. Front. Artif. Intell..

[10] Kouw WM, Loog M (2021). A review of domain adaptation without target labels. IEEE Trans. Pattern Anal. Mach. Intell..

[11] van der Maaten, L. & Hinton, G. Visualizing data using t-SNE. *J. Mach. Learn. Res.* **9**, 2579–2605. http://jmlr.org/papers/v9/vandermaaten08a.html (2008).

[12] Selvaraju, R. R. *et al.* Grad-CAM: Visual explanations from deep networks via gradient-based localization. In *2017 IEEE International Conference on Computer Vision (ICCV)*. 618–626. 10.1109/ICCV.2017.74 (2017).

[13] Que, Q. *et al.* CardioXNet: Automated detection for cardiomegaly based on deep learning. In *2018 40th Annual International Conference of the IEEE Engineering in Medicine and Biology Society (EMBC)*. 612–615. 10.1109/EMBC.2018.8512374 (IEEE, 2018). 10.1109/EMBC.2018.8512374 30440471

[14] Jafar A (2022). CardioNet: Automatic semantic segmentation to calculate the cardiothoracic ratio for cardiomegaly and other chest diseases. J. Pers. Med..

[15] Lee MS (2021). Evaluation of the feasibility of explainable computer-aided detection of cardiomegaly on chest radiographs using deep learning. Sci. Rep..

[16] Ronneberger, O., Fischer, P. & Brox, T. U-Net: Convolutional networks for biomedical image segmentation. In *Medical Image Computing and Computer-Assisted Intervention – MICCAI 2015* (Navab, N., Hornegger, J., Wells, W. M. & Frangi, A. F. eds.). Vol. 9351. 234–241. 10.1007/978-3-319-24574-4_28 (Springer, 2015).

[17] Tang, Y.-B., Tang, Y.-X., Xiao, J. & Summers, R. M. XLSor: A robust and accurate lung segmentor on chest X-rays using criss-cross attention and customized radiorealistic abnormalities generation. In *Proceedings of The 2nd International Conference on Medical Imaging with Deep Learning* (Cardoso, M. J. *et al.* eds.) Vol. 102. *Proceedings of Machine Learning Research*. 457–467. http://proceedings.mlr.press/v102/tang19a/tang19a.pdf (PMLR, 2019).

[18] Saiviroonporn P (2022). A clinical evaluation study of cardiothoracic ratio measurement using artificial intelligence. BMC Med. Imag..

[19] Sogancioglu E (2020). Cardiomegaly detection on chest radiographs: Segmentation versus classification. IEEE Access.

[20] Bortsova, G., Dubost, F., Hogeweg, L., Katramados, I. & de Bruijne, M. Semi-supervised medical image segmentation via learning consistency under transformations. In *Medical Image Computing and Computer Assisted Intervention – MICCAI 2019* (Shen, D. *et al.* eds.) . 810–818. 10.1007/978-3-030-32226-7_90 (Springer, 2019).

[21] Wang H, Gu H, Qin P, Wang J (2022). U-shaped GAN for semi-supervised learning and unsupervised domain adaptation in high resolution chest radiograph segmentation. Front. Med..

[22] Brioso, R. C., Pedrosa, J., Mendonça, A. M. & Campilho, A. Semi-supervised multi-structure segmentation in chest X-ray imaging. In *2023 IEEE 36th International Symposium on Computer-Based Medical Systems (CBMS)*. 814–820. 10.1109/CBMS58004.2023.00325 (2023).

[23] Ouali, Y., Hudelot, C. & Tami, M. Semi-supervised semantic segmentation with cross-consistency training. In *2020 IEEE/CVF Conference on Computer Vision and Pattern Recognition (CVPR)*. 12671–12681. 10.1109/CVPR42600.2020.01269 (IEEE Computer Society, 2020).

[24] Shiraishi J (2000). Development of a digital image database for chest radiographs with and without a lung nodule. Am. J. Roentgenol..

[25] van Ginneken B, Stegmann MB, Loog M (2006). Segmentation of anatomical structures in chest radiographs using supervised methods: A comparative study on a public database. Med. Image Anal..

[26] Ullah I (2023). A deep learning based dual encoder-decoder framework for anatomical structure segmentation in chest X-ray images. Sci. Rep..

[27] Ghali R, Akhloufi MA (2023). Vision transformers for lung segmentation on CXR images. SN Comput. Sci..

[28] Bustos A, Pertusa A, Salinas J-M, de la Iglesia-Vayá M (2020). PadChest: A large chest X-ray image dataset with multi-label annotated reports. Med. Image Anal..

[29] Demner-Fushman D (2016). Preparing a collection of radiology examinations for distribution and retrieval. J. Am. Med. Inf. Assoc..

[30] Demner-Fushman D, Antani S, Simpson M, Thoma GR (2012). Design and development of a multimodal biomedical information retrieval system. J. Comput. Sci. Eng..

[31] Wang, X. *et al.* ChestX-ray8: Hospital-scale chest X-ray database and benchmarks on weakly-supervised classification and localization of common thorax diseases. In *Proceedings of the IEEE Conference on Computer Vision and Pattern Recognition (CVPR)*. 2097–2106. https://openaccess.thecvf.com/content_cvpr_2017/html/Wang_ChestX-ray8_Hospital-Scale_Chest_CVPR_2017_paper.html (2017).

[32] Laine, S. & Aila, T. Temporal ensembling for semi-supervised learning. In *5th International Conference on Learning Representations, ICLR 2017, Toulon, France, April 24–26, 2017, Conference Track Proceedings*. https://openreview.net/forum?id=BJ6oOfqge (OpenReview.net, 2017).

[33] Ma S, Song C (2023). Semi-supervised drivable road segmentation with expanded feature cross-consistency. Appl. Sci..

[34] Bashir RMS, Qaiser T, Raza SEA, Rajpoot NM (2024). Consistency regularisation in varying contexts and feature perturbations for semi-supervised semantic segmentation of histology images. Med. Image Anal..

[35] Tompson, J., Goroshin, R., Jain, A., LeCun, Y. & Bregler, C. Efficient object localization using convolutional networks. In *2015 IEEE Conference on Computer Vision and Pattern Recognition (CVPR)*. 648–656. 10.1109/CVPR.2015.7298664 (IEEE Computer Society, 2015).

[36] Zuiderveld, K. *Graphics Gems IV*. Chap. Contrast Limited Adaptive Histogram Equalization. 474–485. https://dl.acm.org/doi/10.5555/180895.180940 (Academic Press Professional, Inc., 1994).

[37] Woo, S., Park, J., Lee, J.-Y. & Kweon, I. S. CBAM: Convolutional block attention module. In *Proceedings of the 15th European Conference on Computer Vision (ECCV)*. 3–19. 10.1007/978-3-030-01234-2_1 (Springer, 2018).

[38] Kingma, D. P. & Ba, J. Adam: A method for stochastic optimization. In *Proceedings of the 3rd International Conference on Learning Representations*. https://arxiv.org/abs/1412.6980 (2015).

[39] Abadi, M. *et al.* Tensorflow: A system for large-scale machine learning. In *12th USENIX Symposium on Operating Systems Design and Implementation (OSDI 16)*. 265–283. https://www.usenix.org/system/files/conference/osdi16/osdi16-abadi.pdf (2016).

[40] Chollet, F. *Keras* (2015). https://keras.io Accessed 27 July 2023.

[41] Pedregosa, F. *et al.* Scikit-learn: Machine learning in Python. *J. Mach. Learn. Res.* **12**, 2825–2830. https://www.jmlr.org/papers/volume12/pedregosa11a/pedregosa11a.pdf (2011).

[42] Szegedy, C., Vanhoucke, V., Ioffe, S., Shlens, J. & Wojna, Z. Rethinking the inception architecture for computer vision. In *2016 IEEE Conference on Computer Vision and Pattern Recognition (CVPR)*. 2818–2826. 10.1109/CVPR.2016.308 (2016).

[43] Russakovsky O (2015). ImageNet large scale visual recognition challenge. Int. J. Comput. Vis..

[44] Krizhevsky, A., Sutskever, I. & Hinton, G. E. ImageNet classification with deep convolutional neural networks. In *Proceedings of the 25th International Conference on Neural Information Processing Systems*(Pereira, F., Burges, C. J., Bottou, L. & Weinberger, K. Q. eds.). Vol. 25. 1097–1105. https://proceedings.neurips.cc/paper/2012/file/c399862d3b9d6b76c8436e924a68c45b-Paper.pdf (Curran Associates, Inc., 2012).

[45] Lin, T., Goyal, P., Girshick, R., He, K. & Dollar, P. Focal loss for dense object detection. In *2017 IEEE International Conference on Computer Vision (ICCV)*. 2999–3007. 10.1109/ICCV.2017.324 (IEEE Computer Society, 2017).

[46] Jiao, R., Zhang, Y., Ding, L., Cai, R. & Zhang, J. Learning with limited annotations: A survey on deep semi-supervised learning for medical image segmentation. https://arxiv.org/pdf/2207.14191.pdf. arXiv: 2207.14191 (2022). 10.1016/j.compbiomed.2023.107840 38157773

